# Left ventricular hypertrophy and mortality in ethnic minority groups in the UK: e-ECHOES study

**DOI:** 10.1097/HJH.0000000000003561

**Published:** 2023-09-14

**Authors:** Eduard Shantsila, Alena Shantsila, Nefyn Williams, Gregory Y.H. Lip, Paramjit S. Gill

**Affiliations:** a Department of Primary Care and Mental Health, University of Liverpool; b Liverpool Centre for Cardiovascular Science at University of Liverpool, Liverpool John Moores University and Liverpool Heart and Chest Hospital, Liverpool, UK; c Department of Clinical Medicine, Aalborg University, Aalborg, Denmark; d Academic Unit of Primary Care Warwick Medical School, University of Warwick Coventry, UK

**Keywords:** echocardiography, hypertension, left ventricular hypertrophy, mortality

## Abstract

**Objectives:**

Hypertension is the key modifiable cardiovascular risk factor but is underdiagnosed, and its scale in South Asian and African-Caribbean communities is unknown. Left ventricular hypertrophy (LVH) is a measure of target organ damage in uncontrolled hypertension. The study assesses LVH prevalence in South Asian and African-Caribbean communities and its impact on mortality.

**Method:**

This study is based on the large prospective UK community Ethnic-Echocardiographic Heart of England Screening Study (E-ECHOES, age ≥45 years). Left ventricular mass index (LVMI) was calculated using echocardiography to establish LVH. The predictive value of LVH all-cause and cardiovascular mortality was assessed using Cox regression.

**Results:**

The study included 3200 South Asians (age 59 ± 10 years, 52% women, 45% had a history of hypertension, 5.8 ± 1.0-year follow-up). LVH was found in 1568 (49%), of whom 45% did not have hypertension diagnosis. On Cox regression, LVH was independently associated with all-cause mortality [hazard ratio 1.38, 95% confidence interval (95% CI) 1.01–1.88], cardiovascular mortality (hazard ratio 2.64, 95% CI 1.21–3.73). The projected overall hypertension prevalence was 82%, undiagnosed hypertension prevalence 37%. The study included 1858 African-Caribbeans (age 62 ± 12, 45% women, 45% had history of hypertension, 5.1 ± 0.9-year follow-up). LVH was found in 1186 (64%), of whom 32% did not have hypertension diagnosis. LVH was borderline associated with all-cause mortality (hazard ratio 1.57, 95% CI 1.01–2.44), but not cardiovascular mortality (hazard ratio 1.82, 95% CI 0.80–4.16). The projected overall hypertension prevalence was 78.5%, and undiagnosed hypertension prevalence was 20.8%.

**Conclusion:**

UK South Asians and African-Caribbeans have a high prevalence of hypertension, which is often underdiagnosed and poorly controlled.

## INTRODUCTION

Ethnic minority groups, including people of South-Asian (i.e. Indian, Pakistani, Bangladeshi) and African-Caribbean origin, suffer from high cardiovascular morbidity and mortality, contributed by health inequalities. Hypertension is the key modifiable cardiovascular risk factor but is underdiagnosed, and the scale of the problem in these communities is unknown [1]. Left ventricular hypertrophy (LVH) is a measure of target organ damage related to uncontrolled hypertension and can be accurately assessed by echocardiography.

UK South Asians have increased cardiovascular morbidity compared with white people [2]. The difference is contributed by a higher prevalence of cardiovascular risk factors, such as hypertension or type 2 diabetes mellitus [3–5]. The CALIBER platform demonstrated the youngest age of cardiovascular disease in South Asians among all ethnic groups, especially in South Asian women [6]. Although the impact of high diabetes prevalence on cardiovascular risk in South Asians is well recognized, the role of hypertension is less known. This partially reflects the possibility of opportunistic screening for diabetes (i.e. using HbA1c) during blood tests for both acute and routine presentations, while hypertension could be more challenging to diagnose. Moreover, it is possible that blood pressure (BP) thresholds used for diagnosis of hypertension in the white population may not be optimal for South Asians, similar to thresholds for obesity and waist circumference) [7].

Hypertension is highly prevalent among African--Americans, with less effective management is well documented in this ethnic group, leading to higher rates of strokes and mortality [8,9]. UK African-Caribbeans represent a significant ethnic minority group, but scarce data exist on community-level hypertension diagnosis and control.

Ethnic-Echocardiographic Heart of England Screening Study (E-ECHOES) is the largest UK echocardiographic observational study to assess the prevalence of heart failure in ethnic minority groups in the UK. This study documented the population-level prevalence of LVH as a recognized target organ damage secondary to raised BP. The study made every effort to engage a maximum number of patients, for example, by conducting echocardiography in the community, providing interpreters and choosing the sex of the examining researchers. It managed to recruit 50% of eligible patients [10].

The study further tests the impact of LVH on overall and cardiovascular mortality and explores office BP thresholds associated with the presence of LVH and mortality excess.

## MATERIALS AND METHODS

This study is based on the large prospective UK community Ethnic-Echocardiographic Heart of England Screening Study (E-ECHOES) [11]. Echocardiographic data were used to calculate the left ventricular mass index (LVMI) following recommendations of the British Society of Echocardiography [12]. LVH was established as increased LVMI. The predictive value of LVH overall and cardiovascular mortality was assessed using logistic regression (univariate and adjusted for age and sex).

In brief, E-ECHOES was a cross-sectional population survey of a sample of South Asians (i.e. originating from India, Pakistan or Bangladesh) and African Caribbean people aged at least 45 years. The participants were recruited between September 2006 and August 2009 from 20 primary care centres in Birmingham, UK. Potential participants were invited by letter, with a reminder and telephoned up to three times. The evaluation included an interview-administered questionnaire, physical examination, ECG and echocardiogram. Hypertension diagnosis was self-reported during patient interviews and was confirmed with practice medical notes. The study team informed the participants’ GP about cases of raised BP. The study analyses of the prevalence of undiagnosed hypertension were based on the awareness of hypertension diagnosis before the study. The research team was trained at the start of the project on administering the questionnaire and performing a physical examination, ECG and echocardiogram. The team followed written protocols that included several quality control checks, including re-reporting by senior cardiologists of all abnormal echocardiograms and a sample of those reported as normal.

This study complies with the Declaration of Helsinki, and the Walsall Local Research Ethics Committee reviewed and approved the protocol (05/Q2708/45). All participants provided verbal and written consent.

### Statistical analysis

Continuous data are presented as median and interquartile ranges and compared using the Mann--Whitney test. Categorial data are presented as counts (%) and compared using the chi-square test. Univariate and multivariate logistic regression was used to establish predictors of LVH (significant predictors from univariate analysis were used to build the multivariate model). Univariate and multivariate (adjusted for age and sex) Cox regression and Kaplan--Meier plots were used to describe the predictive value of LVH for all-cause and cardiovascular mortality. Statistical analyses were done using Python 3.10 (Pandas, Numpy, Scipy and Statsmodels, matplotlib.pyplot and lifelines libraries).

## RESULTS

### South Asians

The study included 3200 people of South Asian origin who had LVMI (age 59 ± 10 years, 52% women, 93% of recruited). There were 182 deaths (rate 1.0%/year), including 64 cardiovascular deaths (rate 0.3%/year), during a follow-up of 5.8 ± 1.0 years. LVH, defined as increased LVMI, was found in 1568 (49%) people, including mild LVH in 597 (19%), moderate LVH in 442 (14%) and severe LVH in 529 (17%). There were 1440 (45%) people with a history of hypertension, of whom 863 (60%) had LVH. Among those with LVH, 705 (45%) participants did not have a diagnosis of hypertension. LVH is mostly caused by hypertension and its presence at a population level in people without a history of hypertension is a surrogate of undiagnosed hypertension with target organ damage [13]. On the basis of the assumption that LVH is at least as common in untreated undiagnosed hypertension as in diagnosed hypertension (i.e. 60% as above), the projected total number of patients with hypertension was 2613 (82% of the total population) with 1175 (37% of the total population) with undiagnosed hypertension.

People with LVH were older, had higher SBP and DBP, BMI and waist circumference, and were more likely to have other cardiovascular risk factors (diabetes and raised total cholesterol) (*P* < 0.05 for all) (Table 1). People with LVH were more likely to come to the UK at an older age, and reside in more deprived areas (*P* < 0.05 for both). People with LVH had higher rates of estimated glomerular filtration rate (eGFR) in the chronic kidney disease range (i.e. <60 ml/min per 1.73 m^2^), diastolic dysfunction and higher E/E’ ratio (a surrogate marker of increased myocardial stiffness) (*P* > 0.05 for all). On logistic regression, independent predictors of LVH were a history of hypertension, advanced age, higher SBP, higher waist circumference and residence in a more deprived area (*P* < 0.05 for all) (Table 2). Among participants without known hypertension, people with LVH were older, had higher BMI, waist circumference and SBP and DBP, came to the UK at an older age and lived in more deprived areas (*P* < 0.05 for all), but there was no significant difference in sex or interpreter need (*P* > 0.05) (Table 3).

**TABLE 1:** Demographic and clinical characteristics of people with and without left ventricular hypertrophy

	South Asians					African Caribbeans				
	No LVH		LVH		*P*	No LVH		LVH		*P*
	*n*	Values	Yes	Values		*n*	Values	Yes	Values	
Age, years	1632	54 [49–63]	1568	58 [52–69]	<0.001	672	55 [48–68]	1186	66 [52–74]	<0.001
Male sex, *n* (%)	1632	778 (48%)	1568	773 (49%)	0.38	672	276 (41%)	1186	555 (47%)	0.02
Age moving to the UK (years)	1598	21 [16–30]	1549	23 [17–32]	<0.001	513	20 [14–17]	1040	22 [17–29]	0.001
Interpreter use, *n* (%)	1632	304 (19%)	1568	299 (19%)	0.78	672	8 (1%)	1186	4 (0.3%)	0.06
MDI, score	1632	55 [41–62]	1568	53 [38–61]	0.03	672	58 [46–61]	1186	58 [46–62]	0.14
Hypertension, *n* (%)	1632	577 (35%)	1568	863 (55%)	<0.001	672	268 (40%)	1186	803 (68%)	<0.001
Diabetes, *n* (%)	1632	441 (27%)	1568	562 (36%)	<0.001	672	136 (20%)	1186	358 (30%)	<0.001
Smoking, *n* (%)	1632	368 (23%)	1568	368 (23%)	0.56	672	287 (43%)	1186	518 (44%)	0.72
eGFR <60 ml/min per 1.73 m^2^, *n* (%)	558	11 (2.0%)	526	23 (4.4%)	0.04	270	7 (2.6%)	498	29 (5.8%)	0.07
BMI (kg/m^2^)	1632	27.2 [24.5–30.6]	1568	27.9 [25.2–31.4]	<0.001	672	28.1 [25.2–32.2]	1186	29.4 [26.3–33.3]	<0.001
Waist circumference (cm)	1631	95 [89–103]	1566	99 [91–106]	<0.001	671	94 [85–103]	1185	98 [90–107]	<0.001
SBP (mmHg)	1632	136 [123–148]	1567	142 [130–156]	<0.001	672	138 [128–152]	1186	147 [134–161]	<0.001
DBP (mmHg)	1632	80.5 [74–88]	1567	81.0 [74–89]	0.03	672	81 [74–88]	1186	83 [76–90]	<0.001
HbA1c (mmol/mol)	583	6.1 [5.8–6.7]	534	6.2 [5.8–6.8]	0.03	273	6.0 [5.6–6.5]	481	6.2 [5.8–6.7]	0.001
Cholesterol (mmol/l)	568	4.5 [3.7–5.3]	526	4.3 [3.5–5.0]	<0.001	267	4.7 [3.8–5.3]	474	4.4 [3.6–5.3]	0.04
Diastolic dysfunction, *n* (%)	1562	820 (52%)	1497	1016 (68%)	<0.001	638	329 (52%)	1124	779 (69%)	<0.001
E/e’ ratio	1565	7.6 [6.1–9.6]	1512	8.6 [6.7–11.1]	<0.001	640	7.6 [6.2–9.2]	1136	8.3 [6.6–10.5]	<0.001

BP, blood pressure; eGFR, estimated glomerular filtration rate; HbA1c, haemoglobin A1c; LVH, left ventricular hypertrophy; MDI, multiple deprivation index.

**TABLE 2:** Logistic regression for predictors of left ventricular hypertrophy

	South Asian origin				African-Caribbean origin			
	Univariable OR (95% CI)	*P*	Multivariable OR (95% CI)	*P*	Univariable OR (95% CI)	*P*	Multivariable OR (95% CI)	*P*
Age	1.036 [1.029–1.044]	<0.001	1.019 [1.010–1.028]	<0.001	1.042 [1.033–1.051]	<0.001	1.019 [1.006–1.032]	0.003
Male sex	1.067 [0.929–1.226]	0.36	–	–	1.262 [1.042–1.528]	0.017	1.398 [1.093–1.788]	0.008
MDI	0.995 [0.991–0.999]	0.025	0.994 [0.989–0.998]	0.009	1.003 [0.995–1.010]	0.46	–	–
Age moving to the UK	1.016 [1.010–1.022]	<0.001	1.003 [0.997–1.010]	0.32	1.010 [1.001–1.020]	0.038	1.000 [0.991–1.010]	0.93
Interpreter use	1.029 [0.862–1.228]	0.75	–	–	0.281 [0.084–0.936]	0.039	0.334 [0.086–1.303]	0.11
Hypertension (known)	2.238 [1.942–2.580]	<0.001	1.594 [1.354–1.876]	<0.001	3.161 [2.596–3.847]	<0.001	2.137 [1.657–2.757]	<0.001
Diabetes	1.509 [1.298–1.753]	<0.001	1.040 [0.876–1.234]	0.65	1.704 [1.360–2.135]	<0.001	0.951 [0.726–1.246]	0.72
SBP	1.019 [1.015–1.022]	<0.001	1.014 [1.009–1.019]	<0.001	1.023 [1.018–1.028]	<0.001	1.009 [1.001–1.017]	0.023
DBP	1.008 [1.000–1.014]	0.016	0.993 [0.983–1.003]	0.17	1.014 [1.005–1.023]	0.002	1.004 [0.990–1.019]	0.56
BMI	1.034 [1.020–1.049]	<0.001	1.008 [0.987–1.030]	0.46	1.033 [1.016–1.050]	<0.001	0.996 [0.964–1.030]	0.83
Waist circumference	1.022 [1.016–1.028]	<0.001	1.015 [1.006–1.024]	<0.001	1.021 [1.014–1.029]	<0.001	1.013 [0.998–1.027]	0.09
eGFR	0.997 [0.994–1.000]	0.08	–	–	0.997 [0.993–1.000]	0.08	–	
Smoking	1.053 [0.893–1.242]	0.54	–	–	1.040 [0.859–1.259]	0.69	–	–

BP, blood pressure; eGFR, estimated glomerular filtration rate; MDI, multiple deprivation index.

**TABLE 3:** Characteristics of people without known hypertension by presence of left ventricular hypertrophy

	South Asian origin					African-Caribbean origin				
	No LVH		LVH		*P*	No LVH		LVH		*P*
	*n*	values	*n*	values		*n*	values	*n*	values	
Age (years)	1055	53 [48–59]	705	55 [50–65]	<0.001	404	50 [47–59]	383	52 [48–65]	0.006
Male sex, *n* (%)	1055	505 (48%)	705	333 (47%)	0.24	404	175 (43%)	383	183 (48%)	0.24
Age moving to the UK, years	1029	20 [15–26]	693	22 [17–31]	<0.001	273	19 [11–30]	293	19 [12–30]	0.94
Interpreter use, *n* (%)	1055	179 (17%)	705	136 (19%)	0.24	404	5 (1%)	383	2 (0.5%)	0.49
MDI, score	1055	56 [41–62]	705	51 [38–60]	0.002	404	58 [46–61]	383	58 [46–61]	0.86
SBP (mmHg)	1055	132 [121–142]	705	137 [124–150]	<0.001	404	134 [124–146]	383	140 [128–150]	<0.001
DBP (mmHg)	1055	80 [73–86]	705	81 [75–89]	<0.001	404	81 [74–88]	383	82 [77–90]	0.008
BMI (kg/m^2^)	1055	26.8 [24–30]	705	27.3 [25–31]	0.006	404	27.3 [25–31]	383	28.6 [25–32]	0.01
Waist circumference (cm)	1055	94 [88–102]	705	97 [90–103]	<0.001	404	92 [84–101]	383	94 [86–103]	0.026
Smoking, *n* (%)	1055	237 (22%)	705	162 (23%)	0.86	404	187 (46%)	383	189 (49%)	0.43

BP, blood pressure; LVH, left ventricular hypertrophy; MDI, multiple deprivation index.

**FIGURE 1:**
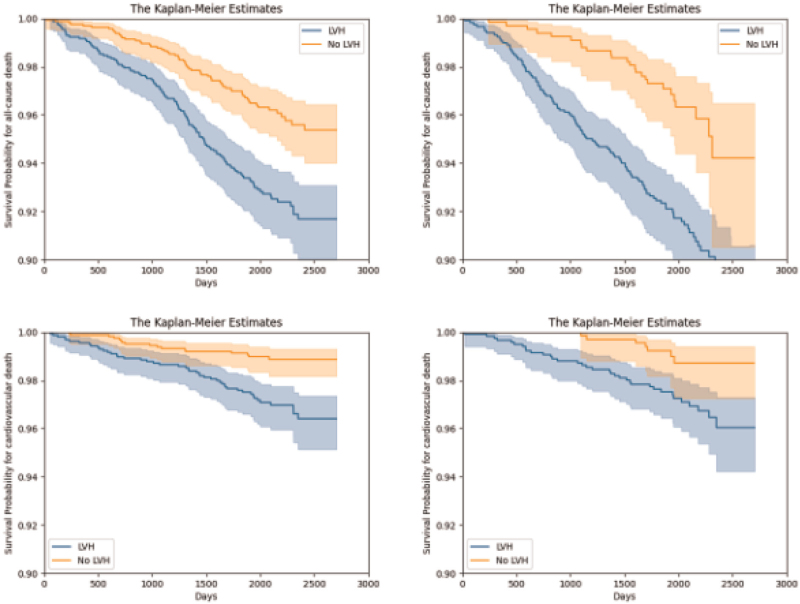
Kaplan--Meier estimates of all-cause and cardiovascular death survival in people with and without left ventricular hypertrophy, Left: survival in South Asians; right: survival in African Caribbeans. LVH, left ventricular hypertrophy.

On Cox regression, LVH was independently associated with all-cause mortality [hazard ratio 1.38, 95% confidence interval (95% CI) 1.01–1.88, *P* = 0.04] after adjustment for age (predictive of death with HR 1.09, 95% CI 1.08–1.11, *P* < 0.005) and sex (predictive of death with hazard ratio 1.48, 95% CI 1.10–1.99, *P* = 0.01) (Fig. 1). Similarly, LVH was independently associated with cardiovascular mortality (hazard ratio 2.15, 95% CI 1.23–3.76, *P* = 0.01) after adjustment for age (predictive of cardiovascular death with hazard ratio 1.08, 95% CI 1.06–1.10, *P* < 0.005) and male sex (predictive of cardiovascular death with hazard ratio 1.69, 95% CI 1.02–2.80, *P* = 0.04).

### African Caribbeans

The study included 1858 people of African-Caribbean origin who had LVMI (age 62 ± 12 years, 55% women, 97% of recruited). There were 134 deaths (rate 1.4%/year), including 42 cardiovascular deaths (rate 0.4%/year), during a follow-up of 5.6 ± 1.1 years. LVH was found in 1186 (64%) people, including mild LVH in 342 (18%), moderate LVH in 282 (15%) and severe LVH in 562 (30%). There were 1071 (58%) people with a history of hypertension, of whom 803 (75%) had LVH. Among those with LVH, 383 (32%) participants did not have a diagnosis of hypertension. On the basis of the assumption that LVH is at least as common in undiagnosed hypertension as in diagnosed hypertension (i.e. 75%), the projected total number of patients with hypertension was 1581 (85% of the total population) with 510 (27% of the total population) with undiagnosed hypertension.

People with LVH were older, had higher SBP and DBP, BMI and waist circumference, included more men and were more likely to have diabetes, but lower total cholesterol) (*P* < 0.05 for all) (Table 1). People with LVH were more likely to come to the UK at an older age (*P* < 0.05). Unlike for South Asians, there was no significant difference in multiple deprivation index, or rates of eGFR in the chronic kidney disease range. People with LVH more often had diastolic dysfunction and a higher E/E’ ratio (*P* > 0.05 for both). On logistic regression, independent predictors of LVH were history of hypertension, advanced age, male sex, higher SBP and waist circumference (*P* < 0.05 for all) (Table 2). Among participants without known hypertension, people with LVH were older, and had higher SBP and DBP, BMI and waist circumference (*P* < 0.05 for all) (Table 3). Very few (*n* = 12, 0.6%) African Caribbean people needed an interpreter and there was no difference in the multiple deprivation index (*P* > 0.05).

On Cox regression, LVH showed borderline significance for independent association with all-cause mortality (hazard ratio 1.57, 95% CI 1.01–2.44, *P* = 0.05) after adjustment for age (predictive of death with hazard ratio 1.09, 95% CI 1.07–1.11, *P* < 0.005) and sex (predictive of death with hazard ratio 1.68, 95% CI 1.19–2.38, *P* < 0.005) (Fig. 1). LVH was not independently associated with cardiovascular mortality (hazard ratio 1.88, 95% CI 0.82–4.27, *P* = 0.13) after adjustment for age (predictive of cardiovascular death with hazard ratio 1.07, 95% CI 1.04–1.10, *P* < 0.005) and male sex (predictive of cardiovascular death with hazard artio 2.49, 95% CI 1.29–4.80, *P* = 0.01).

## DISCUSSION

The principal results of this study indicate that hypertension is highly prevalent in South Asians (82% projected), often underdiagnosed (37% of the total population projected), and inadequately controlled in most people with diagnosed hypertension (60% have LVH). Second, LVH in South Asians was independently associated with a 38% excess in overall mortality and a 2.1-fold increased risk of cardiovascular mortality. The projected prevalence of hypertension in African-Caribbeans is nearly as high (85%) with an even higher rate of LVH (64%). Given the limitations of the BSE reference values for individuals of Afro-Caribbean descent in assessing LVH, the prevalence of LVH may be higher than recorded in this study.

The study provides a unique insight into the population-level rates of diagnosed and nondiagnosed hypertension in the ethnic minority groups in the UK, the prevalence of inadequately controlled hypertension reflected by LVH and its impact on mortality. Although the problem of underdiagnosed hypertension in the UK is widely acknowledged, its scale is hard to assess, especially in disadvantaged and ‘hard-to-reach ethnic minority groups.

The observed prevalence was much higher than 15–20% observed in the predominantly white general population (although our study only included people aged ≥45 years) [14]. Our findings also provide further insights into mechanisms of very high cardiovascular morbidity in this ethnic group. The study calls for an earlier implementation of universal NHS Health Checks in South Asians. The study findings may also reflect the limitations of the traditional diagnosis of hypertension using daytime office measurement, which may miss masked and nocturnal as a cause of LVH [15]. Better awareness of hypertension leads to better hypertension-related outcomes such as risk of stroke and mortality [16].

A major strength of this study is its design or being maximally inclusive by bringing the assessments into the community with the provision of interpreters and choice of researcher's gender, providing as close a population-level evaluation snapshot as realistically possible. The use of LVH as a surrogate of complications.

People who migrated to the UK at an older age were at a higher risk of LVH, and this was not affected the need for interpreter use. This finding may be due to the potential health disadvantages of such people (e.g. supported by higher LVH prevalence in people from more deprived areas). However, the finding may also reflect more extensive epigenetic changes in people longer exposed to living in less prosperous parts of the world.

An interesting observation (based on quartiles of measured BP) was that many people with LVH had BP within the currently accepted BP range. This may indicate the need for tighter BP targets in South Asians and African-Caribbeans to prevent LVH (similar to the established stricter targets for body mass index and waist circumference) [7].

### Limitations

It is possible that some LVH cases were due to reasons other than hypertension (e.g. nondiagnosed hypertrophic cardiomyopathy). Hypertrophic cardiomyopathy has not been listed on reported conditions. It is a rare condition unlikely to impact the results significantly. The analysis used the latest criteria of the British Society of Echocardiography, which have been mainly established on the values of the white population. Still, the study does support the strong prognostic role of the same criteria applied to the South Asian ethnic group. Despite the best efforts, a proportion of eligible patients did not participate in the study.

In conclusion, people of South Asian and African-Caribbean origin in the UK have a very high prevalence of hypertension, which is often underdiagnosed and poorly controlled, leading to target organ damage. In South Asians, LVH leads to excess in overall and cardiovascular mortality. Socioeconomic disadvantages likely contribute to this. The findings call for more effective screening and management of hypertension with lower BP targets.

## ACKNOWLEDGEMENTS

P.G. is part funded by the National Institute for Health Research (NIHR) Applied Research Collaboration West Midlands, and is a NIHR Senior Investigator. The views expressed in this publication are those of the authors and not necessarily those of the NIHR or the UK Department of Health and Social Care.

This work was supported by the British Heart Foundation (PG/05/036), Heart of Birmingham Teaching Primary Care Trust, and through the National Health Service R&D support funding (Primary Care Research Network-Central England).

### Conflicts of interest

None for all authors.
